# Cerebral Fat Embolism Syndrome

**DOI:** 10.5334/jbsr.1781

**Published:** 2019-04-02

**Authors:** Vikram Rao Bollineni, Geert Gelin, Sofie Van Cauter

**Affiliations:** 1 Ziekenhuis Oost-Limburg, Campus St-Jan, Genk, BE

**Keywords:** Fat Embolism Syndrome, DW-MRI, MRI and Star field pattern

## Teaching point

Although cerebral fat embolism syndrome is a clinical diagnosis, Diffusion weighted-MRI (DW-MRI) of the brain showing a “Star Field” pattern of multiple small high-intensity lesions with diffusion restriction supports the diagnosis.

## Report

An 89-year-old male patient was diagnosed with degenerative osteoarthritis of the left knee joint and admitted for unilateral total knee replacement arthroplasty (TKR). Postoperatively (on day 2), the consciousness of the patient rapidly deteriorated with the Glasgow Coma Scale (GCS) falling to 7/15, and he required ventilation and intubation to maintain oxygen saturation level.

**Figure 1:**
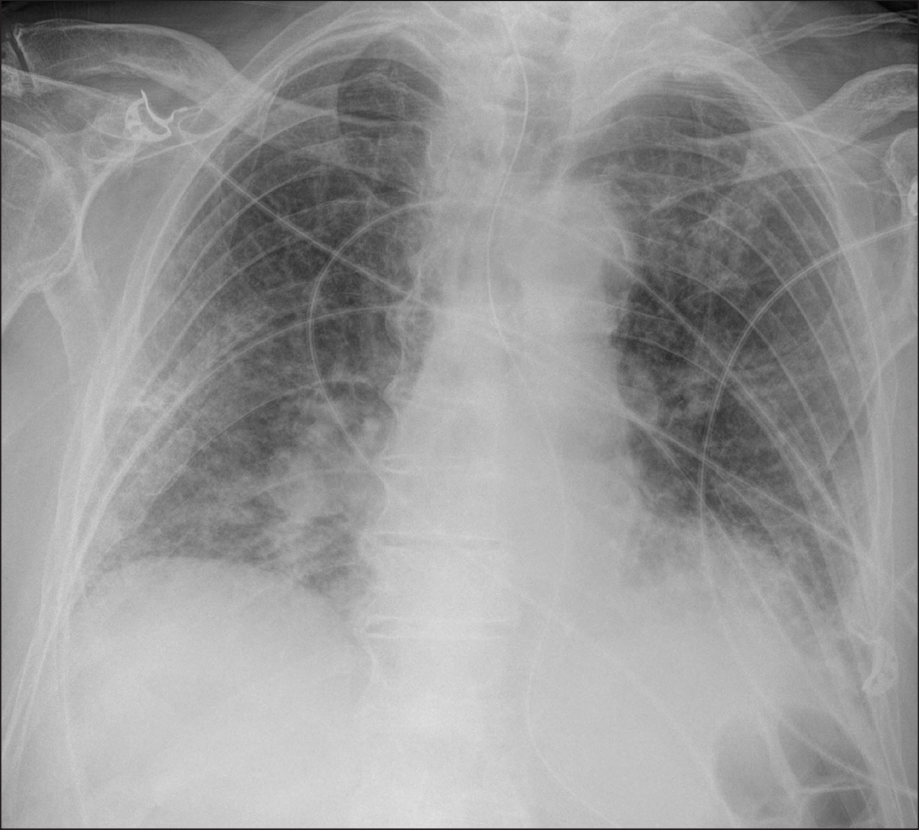
Chest X ray showing bilateral lower lobe consolidations.

**Figure 2:**
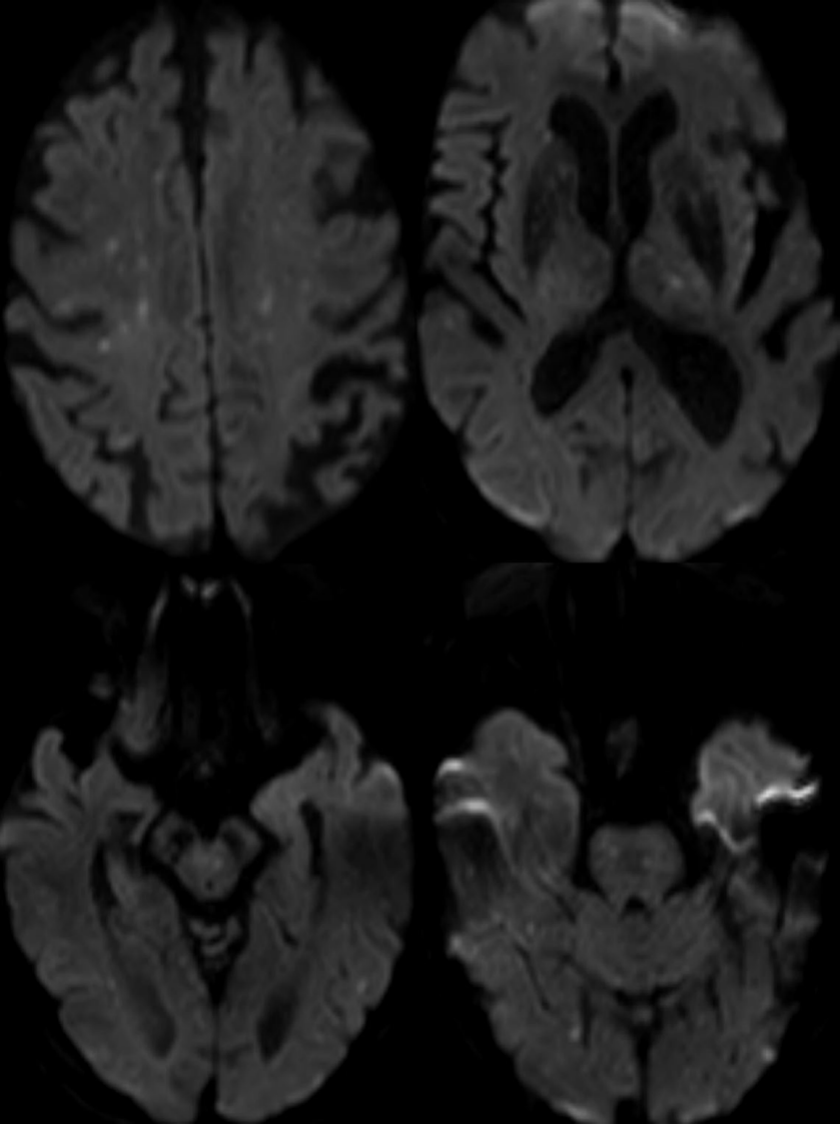
Selected axial DW-MRI images reveal multiple, small, non-confluent foci of diffusion restriction scattered in bilateral cerebral hemispheres, in the basal ganglia, in the brain stem and in the right cerebral peduncle giving a typical “STAR FIELD” appearance.

A chest radiograph showed bilateral lower lobe opacities (Figure 1). Computed tomography (CT) scan of the brain showed no abnormalities. On the sixth day of the postoperative period, the patient underwent a magnetic resonance imaging (MRI) of the brain. On diffusion weighted-MRI (DW-MRI) images, there were multiple small non-confluent hyper-intense diffusion-restricted lesions in the deep white matter of both cerebral hemispheres in a rather symmetric distribution, in the basal ganglia, in the brain stem, and in the right cerebral peduncle. It is this pattern of multiple small high signal intensity DWI lesions in the cerebral white and deep grey matter that has been described as “Star Field” pattern (Figure 2). In view of the clinical content, the diagnosis was made for cerebral fat embolism.

## Comment

The diagnosis of fat embolism syndrome (FES) is a clinical one and related to the release of fat droplets into the systemic circulation after for example an orthopedic intervention. The diagnosis is based on Gurd’s and Wilson’s criteria which require the presence of at least one major and at least four minor criteria (Table 1).

**Table 1:** Gurd’s and Wilson’s criteria.

Major criteria

Petechial rash
Respiratory insufficiency
Cerebral involvement
**Minor criteria**

Tachycardia
Fever
Retinal changes
Jaundice
Renal signs
Thrombocytopenia
Anaemia
High Erythrocyte Sedimentation Rate
Fat macroglobulinemia

The pathophysiology of the fat embolism is not clearly established. Two mechanisms have been postulated [1]. First, mechanical obstruction of the systemic vasculature by fat droplets released from the bone marrow of damaged bone due to high intramedullary pressure during elective orthopedic surgery or trauma, travels through torn veins and lodges in distal capillaries causing mechanical obstruction. Secondly, a biochemical inflammatory response leads to release of free fatty acids and vaso-active substances, which induces local inflammatory response and direct toxicity to the lung and capillary endothelium. This biochemical response alters the stability of chylomicrons that are already present in the bloodstream, which coalesce to form fat globules. These fat globules enter the systemic circulation, resulting in cerebral fat embolism and ischemia.

The diagnosis of fat embolism syndrome is characterized by the classical triad of petechial rash, respiratory distress, and neurological dysfunction. MRI of the brain is a very sensitive technique to confirm the diagnosis of cerebral fat embolism; DWI, in particular, can show the typical “Star Field” pattern with multiple, non-confluent, small hyper-intense foci in the cerebral white matter and deep grey matter in a symmetric distribution.
